# Intramuscular hemorrhage during rehabilitation in a post-stroke patient with vascular Ehlers-Danlos syndrome: a case report and review of spasticity-related muscle injury

**DOI:** 10.3389/fresc.2025.1638656

**Published:** 2025-09-01

**Authors:** Rina Izumi, Koji Hayashi, Mamiko Sato, Tomohisa Yamaguchi, Asuka Suzuki, Yuka Nakaya, Kazumi Ikeda, Masamichi Ikawa, Yasutaka Kobayashi

**Affiliations:** ^1^ Department of Rehabilitation Medicine, Fukui General Hospital, Fukui, Japan; ^2^ Graduate School of Health Science, Fukui Health Science University, Fukui, Japan; ^3^ Department of Neurology, Fukui-Ken Saiseikai Hospital, Fukui, Japan; ^4^ Department of Neurology, University of Fukui, Fukui, Japan; ^5^ Department of Medical Genetics, Faculty of Medical Sciences, University of Fukui, Fukui, Japan; ^6^ Department of Community Health Science, Faculty of Medical Sciences, University of Fukui, Fukui, Japan

**Keywords:** vascular Ehlers-Danlos syndrome, muscle injury, rehabilitation, muscle bleeding, spasticity

## Abstract

We present the first documented case of vascular Ehlers-Danlos syndrome (vEDS) associated with muscle injury in a spastic muscle following a stroke, which occurred during physical therapy. The patient was a 46-year-old male with a family history of subarachnoid hemorrhage (SAH) and aortic dissection, who presented with sudden headache, dysarthria, and left hemiparesis, leading to transport to a nearby hospital. He was diagnosed with arterial dissection and subsequent SAH and cerebral infarction in the right hemisphere using brain computed tomography (CT) and magnetic resonance imaging (MRI). He received treatment with antihypertensive and antiplatelet medications. After five weeks, he was admitted for rehabilitation with moderate left-sided hemiparesis and spasticity. Twenty-six weeks post-onset, while participating in passive hamstring stretching, he experienced sudden pain and swelling in his left thigh. Imaging confirmed hematomas in the biceps femoris and semitendinosus muscles, indicating muscle injury. Clopidogrel was discontinued due to progressive anemia, and the hematoma resolved within five days. He quickly resumed ambulation with increasing independence. One month after the injury, he was discharged home, and subsequent genetic testing at another institution confirmed the diagnosis of vEDS with a pathogenic variant in *COL3A1*. Patients with vEDS are at an increased risk for injuries due to tissue fragility. A stroke can lead to limb spasticity, making spastic muscles more susceptible to injury during sudden stretching, such as passive stretching. This report highlights the need for clinicians to exercise caution when rehabilitating vEDS patients, especially in the absence of established guidelines. Further case reports and clinical evidence are essential to develop comprehensive rehabilitation standards for vEDS.

## Introduction

Spasticity is classically defined as a velocity-dependent increase in muscle tone resulting from exaggerated stretch reflexes (1). However, this definition may be too narrow. A more comprehensive definition characterizes spasticity as disordered sensorimotor control caused by upper motor neuron (UMN) lesions, manifesting as intermittent or sustained involuntary muscle activation (2). This broader definition reflects the complex interplay of factors that contribute to spasticity, including altered sensory feedback, impaired motor control, and changes in spinal cord circuitry (3). In essence, any disease involving UMN lesions can potentially lead to spasticity. Common examples include stroke, spinal cord infarction, demyelinating diseases such as multiple sclerosis, and congenital disorders like cerebral palsy.

Ehlers-Danlos syndrome (EDS) is a group of inherited connective tissue disorders characterized by a range of clinical features including skin hyperelasticity, joint hypermobility, atrophic scarring, and vascular fragility (4, 5). EDS is classically classified into six main types based on the underlying collagen pathology, including classical, vascular, hypermobile, arthrochalasis, kyphoscoliotic, and dermatosparaxis (4).

Among these subtypes, vascular EDS (vEDS), an autosomal dominant disorder, is linked to mutations in the *COL3A1* and/or *COL1A1* genes, which encode for type III and type I collagen, respectively (5). Key clinical features include early-onset arterial rupture, uterine rupture during the third trimester without predisposing factors, and formation of a carotid-cavernous sinus fistula without prior trauma (5). Less prominent features include congenital hip dislocation and spontaneous pneumothorax (5). Diagnosis is supported by a family history and genetic testing.

vEDS is associated with stroke and can lead to spasticity, yet there are no established rehabilitation guidelines and, to our knowledge, no reports of rehabilitation complications have been documented. Here, we present a rare case of vEDS where muscle injury occurred during rehabilitation treatment in a muscle exhibiting spasticity after a stroke.

## Case presentation

**Figure 1:**
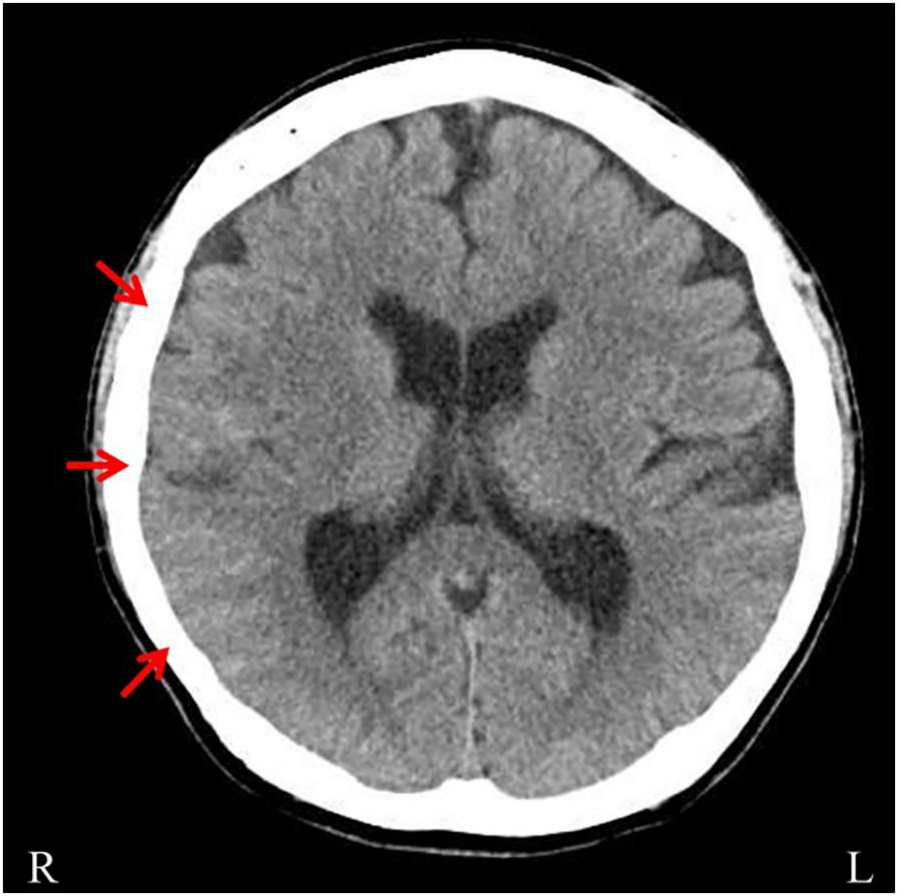
Brain computed tomography (CT). Brain CT demonstrating hyperdense areas in the sulci of the right parieto-occipital lobe (arrows).

**Figure 2:**
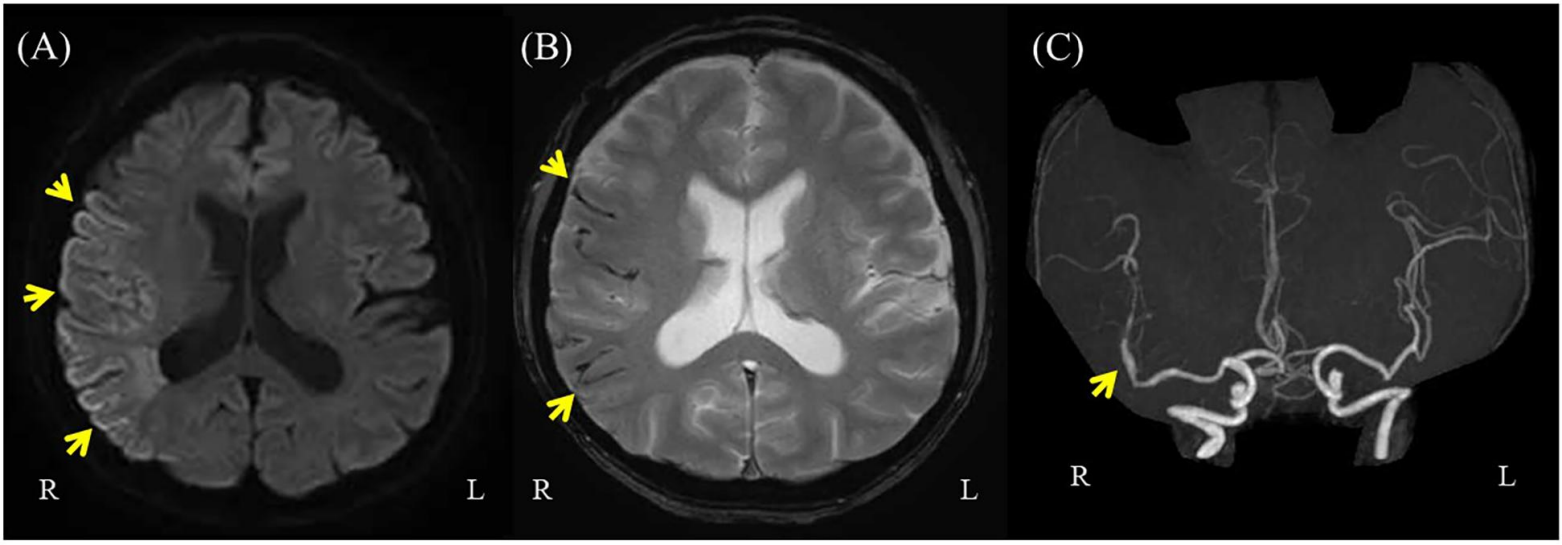
Brain magnetic resonance imaging (MRI) on diffusion-weighted imaging and magnetic resonance angiography (MRA). **(A)** Brain MRI on diffusion-weighted imaging showing hyperintensities in the right middle cerebral artery (MCA) territory (arrows). **(B)** Brain MRI with T2* imaging showing hypointensities on the surface of the brain sulcus. **(C)** Brain MRA showing distal dilatation of the M1 segment and both dilatation and stenosis of the distal M2 segment, exhibiting a “pearl and string sign” that is indicative of arterial dissection (arrow).

A 46-year-old male with a family history of subarachnoid hemorrhage (SAH) in his father and aortic dissection in his nephew developed a sudden headache, dysarthria, and left hemiparesis, and was transported to a previous hospital. His medical history included hypertension and recurrent hemorrhages in the limbs and trunk during light activity. Upon arrival, his vital signs indicated mild disturbed consciousness and high blood pressure. Neurological examination revealed left facial droop, decreased facial sensation, dysarthria, left-sided hemiplegia [the Medical Research Council (MRC) grade 1–2], and reduced sensation. Brain computed tomography (CT) scans revealed hyperdense areas in the sulcus of the right parieto-occipital lobe (Figure 1). Brain magnetic resonance imaging (MRI) showed hyperintensities in the right middle cerebral artery (MCA) territory on diffusion-weighted imaging and hypointensity along the sulci in the right cerebral hemisphere on T2*-weighted imaging (Figures 2A,B). Magnetic resonance angiography (MRA) revealed distal dilatation of the M1 segment and both dilatation and stenosis of the distal M2 segment, exhibiting a “pearl and string sign” that is indicative of arterial dissection (Figure 2C). Cerebral aneurysm was not noted. He was diagnosed with arterial dissection and subsequent SAH and cerebral infarction. He received antihypertensive and antiplatelet drugs including clopidogrel (50 mg/day). After acute phase treatment, he was admitted to our hospital five weeks post-onset for rehabilitation therapy. On admission, his vital signs were unremarkable. He exhibited moderate left-sided hemiparesis with spasticity. The modified Ashworth scale (MAS) scores, a tool used to measure muscle spasticity that assesses resistance felt during passive muscle stretching (ranging from 0, indicating no increase in tone, to 4, indicating rigidity in flexion or extension), were 1+ at the left elbow, hip, and knee joints, and 2 at the ankle. Sensory loss was evident on the left side, with hyperactive deep tendon reflexes and pathological reflexes in the left upper limb. He required minimal assistance to ambulate using handrails and a short leg brace.

**Figure 3:**
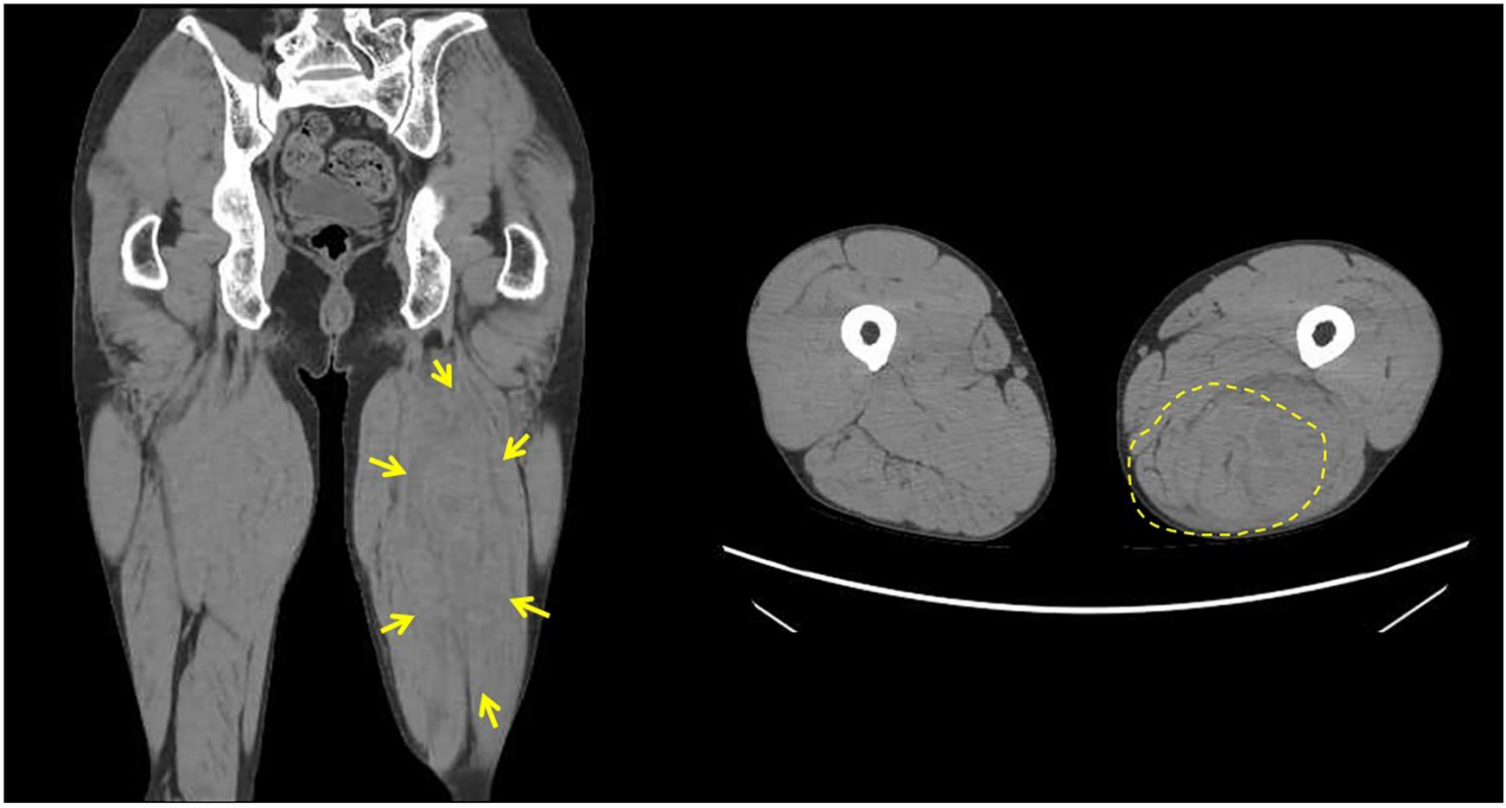
Thigh computed tomography (CT). Thigh CT demonstrating a 177 × 67 mm area of mixed low and high attenuation in the left biceps femoris and semitendinosus muscles.

**Figure 4:**
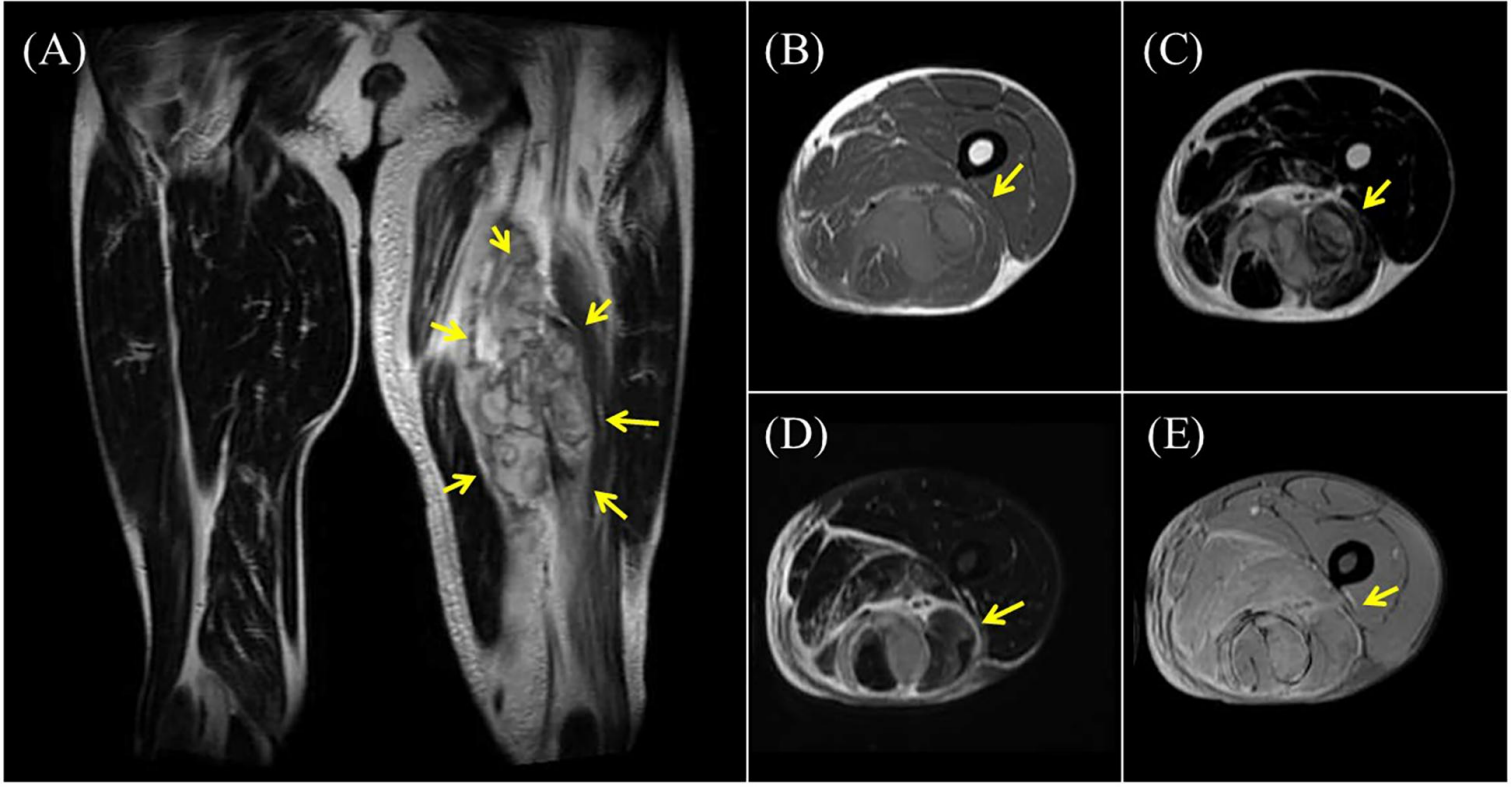
Thigh magnetic resonance imaging (MRI). Thigh MRI showing abnormal signals measuring 156 × 64 × 54 mm in the left biceps femoris and semitendinosus muscles. **(A)** T2-weighted fat-saturated suppression imaging **(B)** T1-weighted imaging **(C,D)** T2-weighted fat-saturated suppression imaging **(E)** T2*-weighted imaging.

Although the patient actively participated in rehabilitation therapy, he experienced sudden pain followed by swelling in his left thigh twenty-six weeks after onset. During hospitalization, his left lower limb muscle tone was frequently observed to increase for unknown reasons, even though this was not reflected in changes in MAS scores. This pattern was also noted in the three days preceding the injury, and it was within this context that the event occurred during a routine passive hamstring stretching session. The procedure was performed by a physical therapist with the patient in the supine position and involved passively flexing the hip to 45 degrees while keeping the knee fully extended. The stretch was applied at the standard intensity commonly used in rehabilitation and did not involve any excessive muscle elongation. Thigh CT and MRI revealed hematomas in the biceps femoris and semitendinosus muscles (Figures 3, 4), suggestive of muscle injury. Blood tests performed immediately after the injury revealed a normal platelet count and prothrombin time-international normalized ratio (PT-INR). Clopidogrel was discontinued due to progressive anemia. In addition, the affected limb was managed with a compression bandage, and the patient received hemostatic agents, including carbazochrome sodium sulfonate and tranexamic acid. The pain gradually improved as the hematoma resolved within five days. He resumed walking exercises on day six, progressively increasing his walking distance until he achieved independent, cane-assisted ambulation within two weeks. One month after the injury, he was discharged home. At another institution, he was confirmed to have a diagnosis of vEDS through genetic testing, which revealed a pathogenic variant in *COL3A1* (detailed information is not available).

## Discussion

We present the first documented case of vEDS associated with muscle injury during physical therapy. The patient was treated with antiplatelet medication for secondary prevention of cerebral infarction, as vEDS had not been diagnosed at the time treatment was initiated. He developed spasticity in the left extremities due to both SAH and cerebral infarction. MAS score for his left leg was assessed as 1+, indicating mild hypertonia. The patient experienced muscle injury and hemorrhage during passive muscle stretching performed at the standard intensity typically used in rehabilitation. His condition improved with the discontinuation of antiplatelet medication, adequate rest, a compression bandage on the affected limb, and the administration of hemostatic agents leading to the resolution of the muscle injury.

Spasticity, characterized by increased muscle tone and exaggerated reflexes, can lead to various structural and functional changes within the affected muscle. In the literature, we have identified four primary mechanisms that underlie these changes associated with spasticity. The first mechanism is muscle fiber degeneration. Repeated spasms and sustained tension can lead to muscle fiber degeneration. This process often involves a reduction in fast-twitch (Type II) fibers, which are responsible for rapid, powerful movements, and an increase in slow-twitch (Type I) fibers, which are more suited for endurance (6, 7). While this shift can improve endurance, it can also reduce overall muscle power and performance. The second mechanism is increased connective tissue. Spasticity is associated with an accumulation of extracellular matrix (ECM) components within the muscle. This increased collagen deposition, often triggered by muscle over-tension and repeated spasms, contributes to muscle stiffness and can impair flexibility (8, 9). This stiffness can, in turn, limit joint range of motion and further hinder function. The third mechanism is fat infiltration and fibrosis. Prolonged spasticity can lead to the replacement of muscle fibers with fat and connective tissue, a process known as fatty infiltration and fibrosis (10, 11). This phenomenon is more pronounced in muscles that are underutilized or chronically spastic. It leads to a significant decline in muscle function. The fourth mechanism is muscle atrophy. Persistent spasticity and inactivity can also cause muscle atrophy. Reduced muscle use leads to a decrease in muscle mass and a loss of the muscle fibers needed for strength and power, significantly impacting muscle performance and contributing to functional limitations (12). In summary, based on these mechanisms, spasticity induces several changes in muscles, diminishing overall power and performance while also causing stiffness, reduced flexibility, and significant declines in muscle function, ultimately contributing to functional limitations.

We summarized six previously reported cases and our case of spasticity accompanied by muscle injury in Table 1 (13–16). Among these cases, three are associated with spinal cord injury, three with cerebral injury, and one with spastic parapresis. While the duration and severity of spasticity varied significantly across cases, a common factor emerged: muscle damage often occurred in individuals with long-standing spasticity during activities involving stretching, such as transfers or passive stretching exercises. The frequency of muscle injuries in patients with spasticity is likely higher than reported, as they often experience external forces on their muscles. This suggests that such injuries may be underreported.

**Table 1:** The summary of present and previous cases of muscle injury with spasticity.

Original disease	Injured muscle	Muscle tone (MAS score)	Years since diagnosis	Mechanism of injury	Antithrombotic therapy	Reference
Brain Injury	Hamstrings	4/4	5 years	Rehabilitation	No	Chua et al. (13)
Multiple Sclerosis	Adductor muscles	4/4	Unknown	Turning to the side	N/A	Patejdl et al. (14)
Spinal Cord Injury (T12)	Semimembranosus	4/4	8 years	Transferring from tractor	No	Carpentier et al. (15)
Spinal Cord Injury (T11)	Semimembranosus	Unknown (AIS-A)	Less than 1 year	Stretching	No	Carpentier et al. (15)
Spinal Cord Injury (T12)	Adductor muscles	Unknown (AIS-A)	17 years	Transferring from wheelchair	No	Carpentier et al. (15)
Hereditary Spastic Paraplegia	Rectus femoris	1/4	30 years	Tripped and extended groin	No	Raes et al. (16)
Cerebral Infarction, Subarachnoid Hemorrhage	Hamstrings	1+/4	5 months	Stretching during rehabilitation	Yes	Current Case

vEDS is primarily caused by a heterozygous mutation in the *COL3A1* gene, which encodes type III collagen. In rare instances, specific heterozygous arginine-to-cysteine substitution mutations in the *COL1A1* gene can also lead to vascular fragility, presenting similar clinical manifestations as vEDS. Type III collagen is a crucial component for the structural integrity of various tissues, including arteries, uterus, bowel, skin, tendons, ligaments, and bones (17, 18). This defect leads to inherent tissue fragility including blood vessels, which is a core characteristic of vEDS (18). While vEDS itself does not cause spasticity, the inherent tissue fragility associated with this condition may significantly exacerbate the vulnerability of muscles already affected by spasticity through a complex interplay of several mechanisms.

In spastic muscle tissue, while ECM compensatorily increases and causes fibrosis, this ECM has been reported to be mechanically inferior to that of normal muscle (19). Thus, spastic muscle can be described as being in a “stiff yet brittle” state due to this proliferative ECM. When the pathology of vEDS is superimposed, this vulnerability may be exacerbated. Type III collagen is essential for the normal fibrillogenesis of type I collagen, a primary component of the ECM (20). Indeed, in homozygous *Col3a1* knockout mice, type I collagen fibrils have been shown to have irregular diameters and disorganized structures (20).

Therefore, it is highly probable that in the spastic muscles of a patient with vEDS, the quantitatively increased ECM also had structural abnormalities at the type I collagen fibril level, stemming from the qualitative defect in type III collagen. As a result, the already fragile ECM of the spastic muscle may have been rendered even more prone to rupture by the underlying pathology of vEDS. Secondly, the vascular fragility inherent in vEDS predisposes patients to arterial dissection, rupture, and increased bleeding tendencies (17, 18). In spastic muscles, sustained contraction can already lead to compromised microcirculation and localized tissue ischemia (9, 11). The underlying vEDS pathology amplifies this risk, increasing susceptibility to micro-hemorrhages or larger hematoma formation within the muscle, further contributing to impaired perfusion and injury (17, 18). This heightened vascular vulnerability means that even minor trauma or stretching can lead to significant bleeding events that might not occur in non-vEDS individuals with spasticity. Therefore, the interplay of compromised ECM integrity, exacerbated fibrosis within the spastic muscle, and heightened vascular fragility collectively renders spastic muscles in vEDS patients particularly susceptible to injury, such as the muscle damage and hemorrhage observed in our case during routine physical therapy. This inherent systemic tissue and vascular fragility results in a wide range of clinical symptoms associated with the syndrome. The literature outlines specific limitations regarding exercise and physical activity for patients with vEDS, recommending the avoidance of high-risk activities such as collision sports (e.g., football), weightlifting, and extreme weight training due to the increased risk of injury, whereas the continuation of low-impact, regular physical activity is advised to preserve both physical health and psychological wellness (21).

Passive stretching of muscles during rehabilitation is typically considered a mild form of exercise; however, in our case, the presence of spasticity due to stroke likely influenced the outcome. This particular muscle injury occurred during a period of increased spasticity, highlighting that fluctuating muscle tone can be a critical risk factor. Additionally, the patient was on antiplatelet medication, which may have contributed to an increased tendency to bleed, resulting in severe intramuscular bleeding. Although genetic testing later revealed vEDS, even if a diagnosis had been made earlier, it would have been difficult to predict muscle damage during rehabilitation therapy, which is generally regarded as a minor exercise. Prior case reports have indicated that injuries to spastic muscles often occur during muscle elongation such as stretching, and our case aligns with this observation, as the muscle injury resulted from passive muscle stretching. For patients with vEDS experiencing spasticity following a stroke, it is crucial to take precautions to prevent intramuscular bleeding when performing passive stretching. Accordingly, in patients with vEDS and post-stroke spasticity, preventive strategies against intramuscular bleeding are essential. In particular, we recommend close monitoring of muscle tone and dynamic adjustment of daily rehabilitation intensity. As there are currently no established guidelines for rehabilitation therapy specific to vEDS, this case report will serve as a significant message for clinicians involved in the rehabilitation of vEDS patients.

This report has several limitations. First, a detailed timeline from stroke onset to rehabilitation admission is absent due to a lack of access to medical records from the previous hospital, preventing a precise delineation of the sequence of events.

Second, a muscle biopsy of the affected site was not performed. Histological analysis could have provided direct evidence of pathological changes, potentially differentiating between alterations due to chronic spasticity (e.g., fibrosis) and those related to the underlying vEDS. Lastly, electrophysiological assessments, including needle electromyography and nerve conduction studies, were not conducted. These tests could have offered a more comprehensive characterization of the underlying muscle pathology and helped to exclude concomitant neuromuscular abnormalities.

## Conclusion

Patients with vEDS are prone to injuries in various body parts due to tissue fragility. Additionally, when a stroke occurs in vEDS patients, it can lead to spasticity in the limbs. It is known that spastic muscles are more susceptible to muscle injury when subjected to sudden stretching, such as during passive stretching. This report serves as an important message for clinicians working with vEDS patients, especially given the lack of clearly established rehabilitation guidelines for this condition. To develop comprehensive rehabilitation standards for vEDS, further accumulation of case reports and clinical evidence is necessary.

## Data Availability

The original contributions presented in the study are included in the article/Supplementary Material, further inquiries can be directed to the corresponding author.
